# Interactions between Fkh1 monomers stabilize its binding to DNA replication origins

**DOI:** 10.1016/j.jbc.2023.105026

**Published:** 2023-07-07

**Authors:** Allan Reinapae, Ivar Ilves, Henel Jürgens, Signe Värv, Kersti Kristjuhan, Arnold Kristjuhan

**Affiliations:** 1 Institute of Molecular and Cell Biology, University of Tartu, Tartu, Estonia; 2 Institute of Technology, University of Tartu, Tartu, Estonia

**Keywords:** DNA replication origin, pre-replicative complex, forkhead transcription factors, Cdc45, *Saccharomyces cerevisiae*, DNA binding proteins

## Abstract

Eukaryotic DNA replication is initiated from multiple genomic origins, which can be broadly categorized as firing early or late in the S phase. Several factors can influence the temporal usage of origins to determine the timing of their firing. In budding yeast, the Forkhead family proteins Fkh1 and Fkh2 bind to a subset of replication origins and activate them at the beginning of the S phase. In these origins, the Fkh1/2 binding sites are arranged in a strict configuration, suggesting that Forkhead factors must bind the origins in a specific manner. To explore these binding mechanisms in more detail, we mapped the domains of Fkh1 that were required for its role in DNA replication regulation. We found that a short region of Fkh1 near its DNA binding domain was essential for the protein to bind and activate replication origins. Analysis of purified Fkh1 proteins revealed that this region mediates dimerization of Fkh1, suggesting that intramolecular contacts of Fkh1 are required for efficient binding and regulation of DNA replication origins. We also show that the Sld3-Sld7-Cdc45 complex is recruited to Forkhead-regulated origins already in the G1 phase and that Fkh1 is constantly required to keep these factors bound on origins before the onset of the S phase. Together, our results suggest that dimerization-mediated stabilization of DNA binding by Fkh1 is crucial for its ability to activate DNA replication origins.

DNA replication is a strictly regulated process that requires the coordinated action of numerous protein complexes. Preparation for DNA synthesis begins already in the G1 phase of the cell cycle, when all potential DNA replication origins are licensed, that is, loaded with pre-replicative complexes (pre-RCs). The core component of the pre-RCs is the minichromosome maintenance complex (MCM), the “molecular motor” of the replicative DNA helicase that is loaded on the origins in an inactive form. Upon the onset of the S-phase, DNA synthesis is initiated from the licensed origins by the sequential action of two kinases. First, the Dbf4-dependent kinase (DDK) phosphorylates the MCM complex, which in turn leads to the recruitment of Sld3, Sld7, and Cdc45 proteins to the pre-RC (1). This step is followed by the Clb5-Cdc28 kinase (CDK)-dependent phosphorylation of multiple targets in the DNA replication machinery, ultimately leading to the activation of the replicative helicase, recruitment of the replicative DNA polymerases, and initiation of DNA synthesis (2, 3, 4). It has been shown that the first step of pre-RC activation—phosphorylation of the MCM complex by DDK and loading of Cdc45—occurs on some replication origins already at the end of the G1 phase, making them ready to initiate DNA synthesis quickly when CDK is activated during the onset of S-phase (5, 6). Therefore, the pre-RC’s ability to efficiently recruit DDK is the critical step that is necessary for the early firing of a DNA replication origin. Although pre-RC phosphorylation by DDK may occur stochastically, at least two mechanisms have evolved to ensure efficient recruitment of DDK to certain replication origins. In budding yeast, the kinetochore protein Ctf19 binds directly to the Dbf4 subunit of DDK and tethers it near the centromeres (7). Therefore, the pre-RCs formed on origins near the centromeric regions are more likely to be phosphorylated by DDK compared to those found in the distal regions. Consequently, these centromere-proximal origins fire early in the S phase (8, 9). Also, the Forkhead family transcription factors have been shown to induce early firing of some replication origins (10, 11). Two possible mechanisms of Forkhead-dependent activation of pre-RCs have been proposed. First, when bound to origins, Forkhead proteins can directly recruit DDK by interacting with its subunit Dbf4 (12); and second, the origin-bound Forkhead proteins can interact with each other to cluster together different origins in the nucleus (13, 14). The latter mechanism increases the local concentration of DNA replication origins, which may lead to more efficient recruitment of DDK than any particular origin could achieve individually.

The Forkhead family transcription factors are widely distributed in eukaryotes, although the number of different Forkhead proteins varies among species. In budding yeast, there are four Forkhead proteins of which Fkh1 and Fkh2 are partially redundant. The primary role of Fkh1 and Fkh2 proteins is to regulate the expression of *CLB2* cluster genes in the G2/M phase (15, 16, 17, 18). In addition, both proteins can also induce the early firing of a subset of DNA replication origins (19). Fkh1 and Fkh2 proteins contain two distinct domains—a forkhead-associated (FHA) domain and a DNA-binding domain. The FHA domain is a binding module for phospho-threonines, and it mediates several protein–protein interactions of Forkhead proteins (20, 21). The DNA binding domain forms a compact winged-helix fold, which is found in various DNA binding proteins (22, 23, 24, 25). It recognizes the consensus binding sequence RYMAAYA (26) that is found all over the budding yeast genome. However, the mere presence of the consensus sequence is insufficient for the efficient recruitment of Forkhead proteins to the site. A genome-wide study of Forkhead binding loci identified that, from approximately 46,000 Fkh1/2 consensus sequences found in the budding yeast genome, only about 1500 sites were bound by Fkh1 or Fkh2 proteins (27). This suggests that Forkhead proteins may need auxiliary DNA binding factors or multiple consensus sites to assist their specific DNA binding. For example, Forkhead-regulated DNA replication origins contain two precisely oriented and spaced Fkh1/2 binding sites near their ARS sequences that ensure the recruitment of Forkhead proteins to these origins. No rearrangement of these sites is tolerated, and the binding of Forkhead proteins to the origins also depends on the full formation of pre-RC (28).

In this study, we show that the DDK-dependent recruitment of replication factors to Forkhead-regulated origins occurs already in the late G1 phase, and the constant presence of Fkh1 is needed for keeping these factors on origins. We also show that a short region in the Fkh1 C-terminus is essential for the regulation of these origins, but it is dispensable for the functioning of Fkh1 as a regulator of cell cycle progression. Our results suggest that this region is mediating the intra-molecular interactions of Fkh1 proteins, which are required for the early firing of Forkhead-dependent replication origins.

## Results

### Sustained binding of Fkh1 is needed for the recruitment of DDK-dependent factors to the pre-RC

To reveal how the Forkhead proteins influence the activation of early-firing replication origins, we first determined the factors that were recruited to the Forkhead-regulated origin *ARS607* in G1-arrested cells. To distinguish Forkhead-dependent and -independent recruitment of replication factors, we used strains carrying an additional copy of *ARS607* with a mutated Fkh1/2 site in an ectopic *VPS13* locus. The *ARS607-fkh-3′-mut* origin is competent for the formation of pre-RC and the initiation of DNA synthesis, but it does not bind Forkhead factors due to a single nucleotide substitution in its distal Fkh1/2 binding site. Consequently, this origin has lost its early-firing properties and initiates DNA synthesis in the late S phase (19). Thus, in these strains, two essentially identical *ARS607* origins were present: wild-type *ARS607* in its native locus and *ARS607-fkh-3′-mut* in the *VPS13* locus, representing Forkhead-binding and non-binding origins, respectively. As a control, we also determined the presence of replication factors in a late-firing and Forkhead-independent *ARS522*, which was not expected to recruit additional replication factors in the G1 phase.

**Figure 1:**
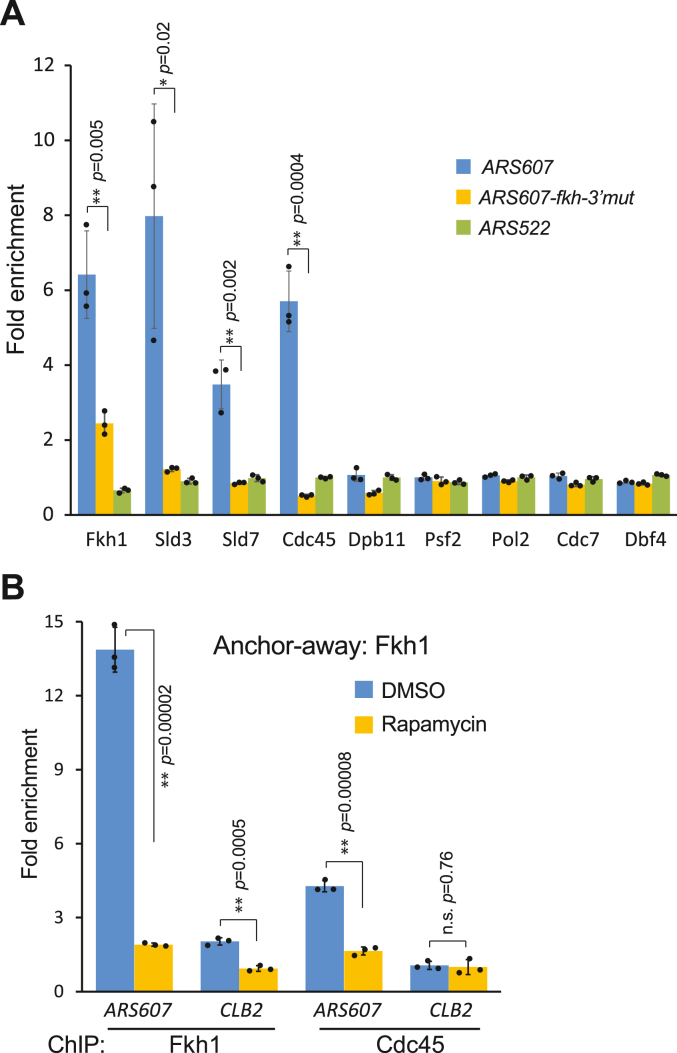
**Recruitment of DNA replication factors to ARS607 in G1-arrested cells.***A*, the strains expressing epitope-tagged replication factors were arrested in G1 with α-factor and the presence of the indicated proteins was determined by ChIP assay followed by qPCR on *ARS607*, *ARS607* with a mutated 3′ Fkh1/2 binding site (*ARS607-fkh-3′mut*), and a late replicating origin *ARS522*. *B*, the Fkh1 anchor-away strain was arrested in G1, treated with rapamycin for the depletion of Fkh1, or with DMSO for control, and the presence of epitope-tagged Fkh1 and Cdc45 proteins was detected on *ARS607*. The *CLB2* promoter region was used as a control for Fkh1 binding to a replication-unrelated locus. The graphs show the fold enrichment of the ChIP signal relative to no antibody control and represent the average of three independent experiments. *Black dots* represent the individual data points; error bars represent the standard deviation; ∗ indicates the *p*-value < 0.05; ∗∗ indicates the *p*-value < 0.01; and n.s., not significant.

On wild-type *ARS607* but not on the origin with a mutated Fkh1/2 binding site, we detected Sld3, Sld7, and Cdc45—all proteins that were expected to be recruited to pre-RC only after phosphorylation of the MCM complex by DDK (Fig. 1*A*). In contrast, Dpb11, Psf2, and Pol2—the factors that require CDK action before association with pre-RC—did not bind origins in G1 (Fig. 1*A*). Interestingly, although the DDK-dependent replication factors were recruited to *ARS607*, neither subunits of the DDK kinase (Dbf4 and Cdc7) were detected on the origin (Fig. 1*A*). This suggests that DDK interaction with Forkhead-regulated origins is transient, although sufficient to trigger the association of Sld3-Sld7-Cdc45 with the pre-RC.

The presence of Sld3-Sld7 and Cdc45 on Forkhead-regulated origins in G1 supports the model that the primary role of Forkhead proteins is to bring DDK to the licensed origins and initiate the loading of Cdc45 to pre-RCs. This model suggests that once the MCM complex is phosphorylated and Cdc45 is loaded to pre-RCs, the presence of Forkhead proteins is not necessary to keep Cdc45 on origins. To test this hypothesis, we used the anchor-away technique (29) to remove Fkh1 from cell nuclei in G1-arrested cells and determined the presence of Cdc45 on a Forkhead-regulated origin. Surprisingly, we saw that upon depletion of Fkh1, also Cdc45 was removed from *ARS607* (Fig. 1*B*), indicating that Fkh1 was constantly needed to keep the Sld3-Sld7-Cdc45 complex on the origin. As expected, the removal of Sld3, Sld7, or DDK from the nucleus also caused the dissociation of Cdc45 from the origin, while the removal of Psf2, which acts in the post-Cdc45 recruitment step of origin activation, did not affect the binding of Cdc45 to the origin (Fig. S1*A*).

### DDK-dependent pre-RC components are not required for Fkh1 binding to the replication origins

We have previously shown that the recruitment of Fkh1 requires fully licensed origins, as disruption of the origin licensing either by mutation of the ARS consensus sequence or inactivation of the Mcm4 protein displaces Fkh1 from the origins (28). This raises the possibility that pre-RC proteins may stabilize Fkh1 binding with replication origins, either *via* direct interactions or by the rearrangement of local chromatin structure. Previous studies indicated that both mechanisms may stabilize the DNA binding of the Forkhead proteins. For example, it has been shown that Fkh2 binds cooperatively with Mcm1 to the *SWI5* promoter, where both factors together ensure proper activation of *SWI5* transcription (30, 31). On the other hand, it has been shown that the nucleosomal structure of DNA replication origins turns into a more closed conformation when the pre-RC formation is disrupted by the inactivation of the ORC complex (32, 33), which in turn can make Fkh1/2 binding sites inaccessible. As the Forkhead-dependent origins are also loaded with the Sld3-Sld7-Cdc45 complex, we tested whether these DDK-dependent factors might be required for the stabilization of Fkh1 binding to the origins. We depleted either Sld3, Sld7, Cdc45, or Pol2 from cell nuclei in G1-arrested cells and determined Fkh1 recruitment to *ARS607* before and after the depletion of pre-RC components. None of these factors influenced the efficiency of Fkh1 binding (Fig. S2), suggesting that the Sld3-Sld7-Cdc45 complex is not required for the stabilization of Fkh1 on origins.

### The C-terminus of Fkh1 is required for the regulation of early replication origins

**Figure 2:**
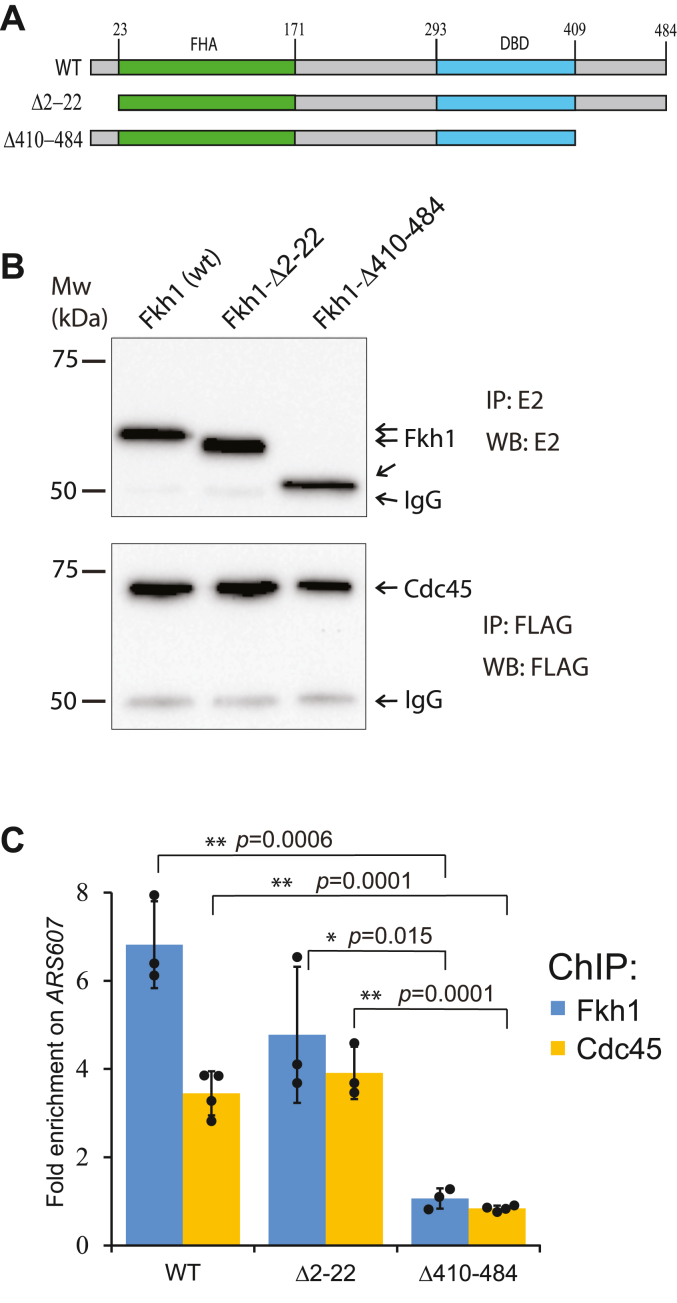
**The C-terminus of Fkh1 is required for binding to replication origins.***A*, the scheme of Fkh1 deletion mutants tested in the ChIP assays. The locations of the *Forkhead-associated domain* (FHA) and *DNA binding domain* (DBD) are indicated in *green* and *blue*, respectively. *B*, the IP-western blot from strains expressing E2-tagged Fkh1 and FLAG-tagged Cdc45 proteins. The proteins were immunoprecipitated from the whole cell extract with either E2 or FLAG antibodies, separated on 10% SDS-PAGE, and blotted with the same antibodies. *C*, strains expressing wild-type Fkh1 or its deletion mutants Δ2-22 or Δ410–484 were arrested in G1, and the presence of Fkh1 and Cdc45 in the *ARS607* locus was determined by ChIP followed by qPCR. The graph shows the fold enrichment of the ChIP signal relative to no antibody control and represents the average of three independent experiments. *Black dots* represent the individual data points; error bars represent the standard deviation; ∗ indicates the *p*-value < 0.05; and ∗∗ indicates the *p*-value < 0.01.

To reveal the functional domains of Fkh1 that were required for the regulation of DNA replication origins, we first tested whether the Fkh1 regions outside of its well-structured FHA and DNA-binding domains were needed to support the early firing of the replication origins. For that, we made the strains expressing either N- or C-terminally truncated Fkh1 proteins in the *fkh2Δ* strain background and confirmed their expression in the cells (Fig. 2, *A* and *B*). Next, we tested whether the mutant Fkh1 proteins could bind to Forkhead-activated DNA replication origins and induce the recruitment of Cdc45. We found that the N-terminally truncated Fkh1 (Δ2-22) was fully competent for binding to Forkhead-regulated origin *ARS607*, and it also recruited Cdc45 to the pre-RC, while the C-terminally truncated protein (Δ410–484) had lost these properties (Fig. 2*C*). These results indicate that the C-terminus of Fkh1 is required for its ability to activate replication origins.

**Figure 3:**
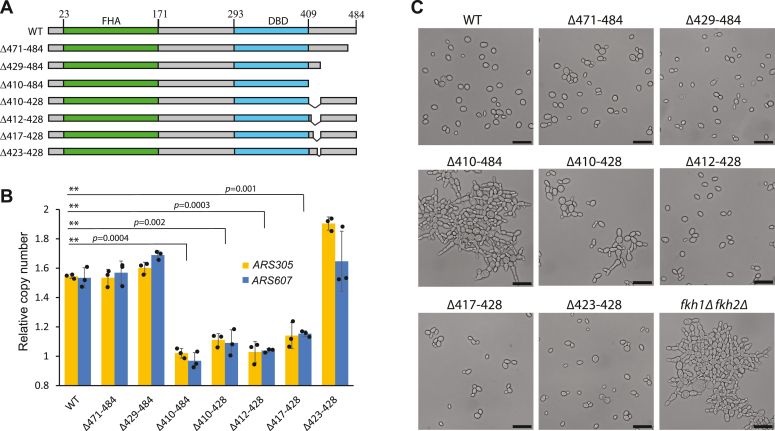
**A short C-terminal region in Fkh1 is required for the activation of replication origins.***A*, the scheme of Fkh1 short C-terminal deletion mutants tested in the DNA-doubling assay. The locations of the *Forkhead-associated domain* (FHA) and *DNA binding domain* (DBD) are indicated in *green* and *blue*, respectively. *B*, the strains expressing different Fkh1 deletion mutants were arrested in G1 and released into S-phase for 120 min in the presence of 200 mM hydroxyurea (HU). The copy number of Forkhead-regulated *ARS305* and *ARS607* relative to the late-replicating telomeric *ARS522* locus was determined by qPCR. The ratio of *ARS305* or *ARS607* to the *ARS522* in G1-arrested cells was arbitrarily set to “1” and the graph shows the change of this ratio after incubation of cells for 120 min in the HU-containing media. The relative copy number “1” indicates that *ARS305* or *ARS607* loci were not replicated, while the relative copy number “2” indicates full replication of the locus. The graphs represent the average result of three independent experiments. *Black dots* represent the individual data points; error bars represent the standard deviation; and ∗∗ indicates the *p-value* < 0.01. *C*, differential interference contrast photographs of the strains expressing the indicated Fkh1 mutants. All strains, except wild type (WT), are also *fkh2Δ*. Scale bar = 20 μm.

To map more precisely the Fkh1 domains required for replication origin regulation, we made additional truncations and internal deletions in the C-terminus of the protein (Fig. 3*A*) and tested their ability to assure early firing of Forkhead-regulated replication origins. First, cells were arrested in G1 and then released into the S phase in media containing 200 mM hydroxyurea. In these conditions, early origins can fire, but activation of the late origins is halted. This leads to the replication of genomic loci located near early origins, while the late-replicating regions remain unduplicated. To reveal the Fkh1 mutants that can support the early firing of origins, we determined the copy-number change in Forkhead-regulated origins *ARS305* and *ARS607* after releasing the strains into the S phase. We found that in contrast to the Δ410–484 mutant, the shorter C-terminal truncations of the Fkh1 protein (Δ471–484 and Δ429–484) were fully competent in the regulation of replication origins (Fig. 3*B*), suggesting that amino acids 410 to 428 were required for this activity. Next, we introduced internal deletions into this region and found that the Fkh1 mutant Δ423–428 was able to activate origins, while all other internal deletion mutants had lost this property (Fig. 3*B*). The shortest inactivating deletion mutant was Δ417–428, emphasizing the importance of this region in the regulation of replication origins. As the Fkh1 mutant Δ423–428 was fully functional in the activation of origins, we propose that the amino acids 417 to 423 are required for Fkh1 to support the early activation of replication origins.

One of the main roles of Forkhead proteins is to regulate the proper expression of G2/M cyclins. In that respect, Fkh1 and Fkh2 are functionally redundant, and the deletion of either gene does not lead to any significant phenotypic changes in yeast. However, the simultaneous deletion of *FKH1* and *FKH2* leads to improper regulation of the ‘*CLB2* cluster’ genes in G2, which in turn leads to defects in cytokinesis, the formation of pseudohyphae, and flocculation of cells in liquid media (15, 17, 34). To test whether the Fkh1 mutants were also defective in the regulation of cell division, we examined their phenotype microscopically. The strains expressing different Fkh1 truncations or internal deletions were grown in liquid media into the mid-log phase, sonicated for the separation of cells, and photographed on a microscope slide. We found that the Fkh1 mutant Δ410–484 displayed the typical *fkh1Δ fkh2Δ* phenotype, and to a lesser extent, a similar phenotype was visible in the *fkh1-Δ410*–*428* strain. All other Fkh1 mutants were indistinguishable from the wild-type strain (Fig. 3*C*). We confirmed these phenotypes by the sedimentation assay, which also indicated flocculation of *fkh1-Δ410*–*484* cells in liquid media (Fig. S3). Together, these results show that a short region in the Fkh1 C-terminus is specifically required for its ability to regulate the early firing of replication origins but not for its functioning as a cell cycle regulator.

### The Fkh1 C-terminal region mediates interactions between Fkh1 monomers

**Figure 4:**
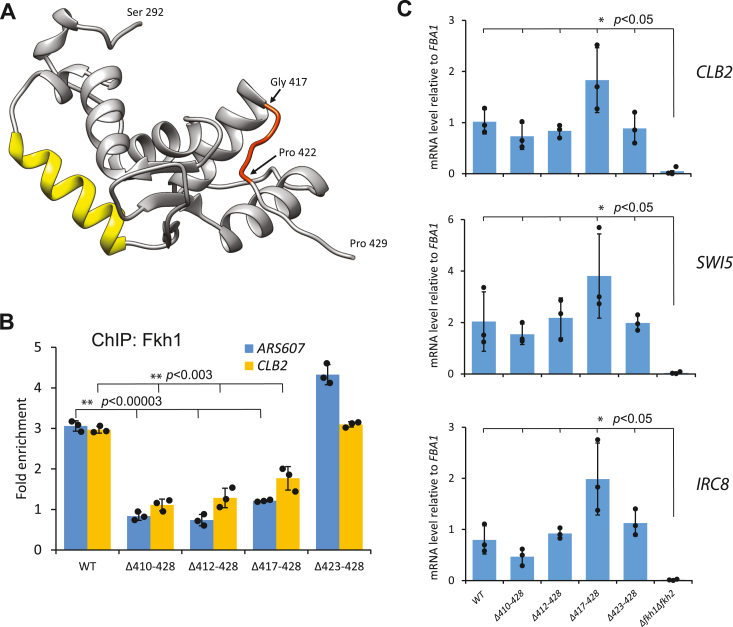
**DNA binding of Fkh1 internal deletion mutants.***A*, the AlphaFold database structure prediction of the Fkh1 DNA binding domain. The region between amino acid residues 292 to 429 of Fkh1 is shown. The DNA-recognition helix is shown in *yellow*, and the region affecting Fkh1 ability to activate early firing of replication origins is shown in *red*. *B*, the binding of Fkh1 small internal deletion mutants to *ARS607* and *CLB2* loci was determined in G1-arrested cells by a ChIP assay followed by qPCR. The graphs show the fold enrichment of the ChIP signal relative to no antibody control and represent the average of three independent experiments. *Black dots* represent the individual data points, error bars represent the standard deviation, and ∗∗ indicates the *p*-value < 0.01. *C*, RT-qPCR analysis of the mRNA levels of *CLB2*, *SWI5*, and *IRC8* genes in strains expressing small internal deletions of Fkh1 in the *fkh2Δ* strain background. The samples were collected 60 min after the release from G1 arrest and represent the cells in the G2/M phase. The cell cycle profiles of the strains are shown in Figure S4. mRNA levels are shown relative to the 1/1000 *FBA1* mRNA level. *Black dots* represent the individual data points; error bars represent the standard deviation; and ∗ indicates the *p*-value < 0.05.

The AlphaFold database prediction of Fkh1 structure indicates that the region we found necessary for activation of replication origins is located immediately after the Fkh1 DNA binding domain, away from the DNA recognition helix (35, 36). Therefore, this region is unlikely to make direct contact with DNA (Fig. 4*A*). To test whether the Fkh1 proteins with deletions inside this region can bind to their genomic target sites *in vivo*, we followed their recruitment to the replication origin *ARS607* and the *CLB2* gene promoter. We found that all tested mutants, except Δ423–428, bound their target loci less efficiently than the wild-type protein (Fig. 4*B*). Interestingly, although the binding of Fkh1 mutants Δ412–428 and Δ417–428 to the *CLB2* locus was reduced compared to wild-type and Δ423–428 proteins, both mutants remained functional in cell cycle regulation as the phenotype of these strains was similar to the wild-type (Figs. 3*C* and S3). This suggested that despite the apparent diminished presence of Fkh1 protein on the promoter loci, Fkh1-regulated gene expression was still functional in these mutant strains. To test directly whether the short internal deletions of Fkh1 affect its ability to regulate its target genes, we measured the mRNA leaves of Forkhead-dependent genes in G2. First, the strains were arrested in G1 with α-factor, and then released synchronously into the S and G2 phases. We collected mRNA samples 60 min after the release from G1, when the cells had reached the G2/M phase (Fig. S4). RT-qPCR analysis of Forkhead-regulated genes *CLB2*, *SWI5*, and *IRC8* indicated that all strains with short internal C-terminal deletions of Fkh1 were able to activate expression of the Forkhead target genes, while the *fkh1Δ fkh2Δ* strain had lost this ability, confirming that all Fkh1 internal deletion mutants retained the cell cycle regulation activity (Fig. 4*C*).

**Figure 5:**
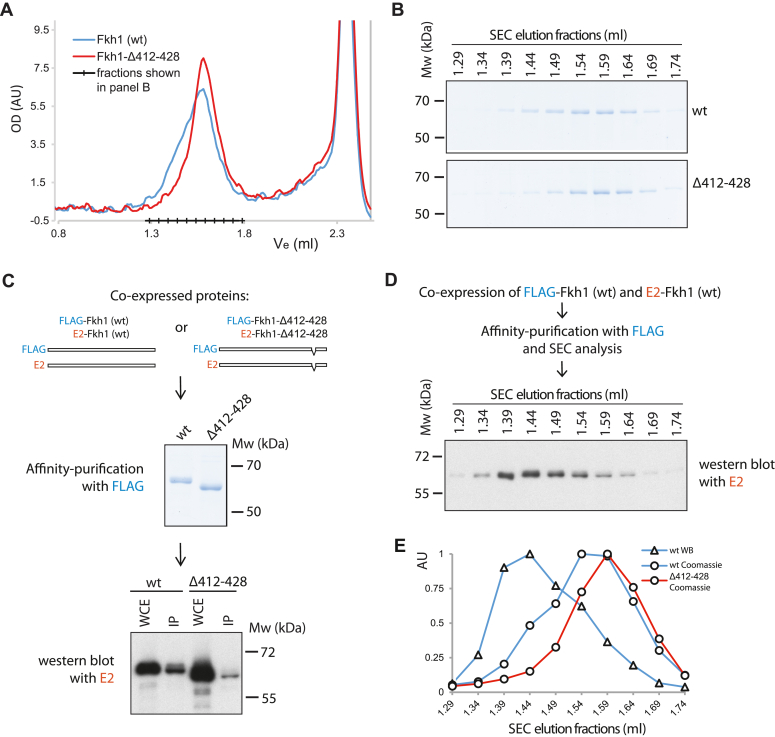
**Dimerization analysis of purified Fkh1 proteins.***A*, size exclusion chromatography (SEC) elution profiles of purified full-length Fkh1 (*blue line*) and Fkh1-Δ412–428 (*red line*) proteins. *B*, full-length Fkh1 and Fkh1-Δ412–428 eluates from the SEC column in the range between 1.29 to 1.79 ml were collected into 50 μl fractions, analyzed on 10% SDS-polyacrylamide gel, and stained with Coomassie Brilliant *Blue*. The starting volumes of each fraction are shown on *top* of the lanes. *C*, whole-cell extract (WCE) from the strains expressing two copies of differently tagged full-length or Δ412–428 Fkh1 proteins was used for affinity purification of FLAG-tagged proteins. The *middle panel* shows the Coomassie Brilliant *Blue*-stained SDS-polyacrylamide gel (10%) of the FLAG affinity-purified proteins. The *lower panel* shows the Western blot of E2-tagged protein in the samples before (WCE) and after (IP) affinity purification with the FLAG affinity resin. *D*, co-expressed FLAG- and E2-tagged full-length Fkh1 proteins were affinity-purified with FLAG and fractionated by SEC, and the presence of co-purified E2-tagged protein in different fractions was determined by western blotting. *E*, quantification of the data from *panels B* and *D* showing the presence of E2-tagged protein predominantly in the ‘shoulder’ fractions of the SEC.

Our previous study revealed that Forkhead-regulated origins require two Fkh1/2 binding sites that are oriented in divergent directions and separated by 72 base pairs. Notably, any disruption of this arrangement, for example by changing the distance between the sites by as little as 5 bp or reversing the directionality of the sites, abolishes Forkhead-dependent regulation of the origins (28). This suggests that on DNA replication origins, two Forkhead proteins bind their consensus sequences in a conformation that may allow them to stabilize each other's interactions with the origin. To test whether the Fkh1 region between amino acids 412 to 428 may modulate its biochemical properties, we affinity-purified the FLAG-tagged full-length and Δ412–428 Fkh1 proteins from the yeast cells and analyzed their elution profiles on size exclusion chromatography (SEC). This method separates proteins and protein complexes based on the size of the molecules in solution, which in turn is determined by the molecular weight as well as the overall shape of the molecules. Although both proteins eluted from the SEC column as a single peak, the elution profile of the full-length protein consistently displayed a “shoulder” toward the larger molecular size, which was never detected in the elution profile of the Δ412–428 mutant during several independent chromatography runs (Figs. 5*A* and S5). The SDS-PAGE analysis of the eluted fractions showed the presence of a single major protein band in all the fractions of the full-length Fkh1 peak, including the “shoulder” (Fig. 5*B*), the electrophoretic mobility of which was consistent with the expected molecular weight of Fkh1. Therefore, the presence of the larger size sub-population in the purified full-length Fkh1 preparations suggested that this represents the same protein either folded into an alternative conformation or forming different oligomers, and this property was lost in the Δ412–428 mutant. Next, we analyzed the hydrodynamic properties of the full-length Fkh1 protein in the main peak and the “shoulder” fractions by glycerol gradient sedimentation analysis. We found that in addition to the larger molecule size, the protein from the “shoulder” fractions is also characterized by a higher sedimentation coefficient (S) value, suggesting that it corresponds to the higher oligomeric state of Fkh1. Approximate molecular weight estimations based on the measured Stokes radius (Rs, from SEC analysis) and S values suggested that in the main peak, the Fkh1 protein was monomeric, while the “shoulder” additionally contained the protein in its dimeric form (Fig. S5).

To confirm that the 412 to 428 region can support the dimerization of Fkh1, we co-expressed two Fkh1 proteins tagged either with a FLAG or E2 tag, affinity-purified the FLAG-tagged protein, and analyzed whether the E2-tagged protein co-purified with it. When full-length Fkh1 proteins were co-expressed, the E2-tagged protein was efficiently co-purified with the FLAG-tagged protein, indicating the association between Fkh1 monomers. In contrast, the interaction between Fkh1-Δ412–428 monomers was significantly reduced compared to the wild-type protein (Fig. 5*C*). In addition, when the fractions from the SEC analysis of the FLAG affinity purified full-length Fkh1 were probed with the E2 antibody, the peak of the co-purified E2-tagged Fkh1 was detected in the fractions representing the “shoulder” of the SEC elution profile, indicating that it indeed corresponds to the Fkh1 dimer (Fig. 5, *D* and *E*).

Taken together, we propose that the Fkh1 region located immediately after its DNA binding domain is required for the formation of intermolecular interactions between Fkh1 monomers, and this property is needed for the stabilization of Fkh1 binding to and subsequent activation of DNA replication origins.

## Discussion

Fkh1 and Fkh2 proteins have several functions in yeast cells. Their primary role is to regulate the expression of G2/M cyclins, but they can also activate the firing of a subset of DNA replication origins in the early S phase. The current model proposes that when bound to origins in the G1 phase, the Forkhead proteins recruit DDK to pre-RCs to phosphorylate the MCM complex. This in turn is required for the recruitment of the Sld3-Sld7-Cdc45 complex to pre-RC. When loaded with Cdc45, the origin is ready for the recruitment of the CDK-dependent replication factors when cells enter the S phase. This model suggests that once the MCM complex is phosphorylated by DDK and Cdc45 is recruited to the pre-RC, the Forkhead proteins have no active role in keeping the origin in early-firing status. We tested this hypothesis by removing Fkh1 from the nucleus in the G1-arrested cells and determined whether Cdc45 remains bound to the pre-RC in these conditions. Surprisingly, we saw that the removal of Fkh1 from pre-RC also led to the displacement of Cdc45 (Fig. 1*B*), indicating that either the Forkhead proteins have a more direct role in stabilizing the Sld3-Sld7-Cdc45 interaction with pre-RC or the turnover of MCM phosphorylation is relatively fast and the Forkhead-dependent recruitment of DDK is continuously needed to keep it phosphorylated. Alternatively, other cellular processes, engaged with DNA and chromatin, may disassemble the whole pre-RC from the origin, and therefore, the complex has to be reloaded repeatedly in G1. Indeed, we have shown that active transcription in the origin region displaces pre-RCs, which are reassembled upon transcription repression (37). The vast majority of budding yeast DNA replication origins are located in the intergenic regions and overlap largely with the gene promoters. Therefore, the origins are constantly under transcriptional stress and may require reloading of pre-RCs several times in G1. Hence, the disappearance of Cdc45 from origins upon depletion of Fkh1 may reflect the fact that the Forkhead-dependent reloading of Sld3-Sld7-Cdc45 is also hampered in these circumstances. We also did the reciprocal experiment and tested whether the recruitment of Fkh1 to pre-RC does depend on the presence of the Sld3-Sld7-Cdc45 complex on origin. We found none of these proteins to be a prerequisite for the binding of Fkh1 to the origin, which also confirms that the binding of Fkh1 precedes the recruitment of the Sld3-Sld7-Cdc45 complex (Fig. S2).

To determine the domains that are essential for Fkh1 to activate replication origins, we made a series of Fkh1 deletion mutants and tested their ability to support the early firing of origins and the normal progression of the cell cycle. We found that a short region in the C-terminal part of the Fkh1 protein (amino acid residues 417–423) was specifically required for the activation of replication origins but not for the regulation of the cell cycle. Next, we tested whether these mutants could bind the replication origin *ARS607* and the promoter region of the *CLB2* gene. Although Fkh1 binding was reduced in both loci, it affected only the activation of DNA replication origins but had no consequences for normal cell cycle regulation or the induction of Forkhead target genes in G2 (Figs. 3*C* and 4C). The mechanistic reasons for this distinction are unclear, but we propose that even the transient interaction of Fkh1 with its target promoters may be sufficient to trigger gene expression, while the activation of DNA replication origins requires a more permanent presence of Fkh1 to ensure the maintenance of origin-bound replication factors in late G1.

To understand the mechanisms supporting the stable binding of Forkhead proteins to the replication origins, we purified the full-length and Δ412–428 Fkh1 proteins and studied their biochemical properties. We detected only the monomeric form of the Δ412–428 protein, while the full-length protein was able to establish unstable dimers *in vitro* (Figs. 5 and S5). As cell cycle progression was not compromised in the Fkh1 Δ412–428 strain, it suggests that the dimerization of Fkh1 is not critical for all of its functions. It is possible that monomeric Fkh1 protein can bind its consensus sites with low affinity, and this interaction can be further stabilized by the interactions between Fkh1 monomers or by the interactions with other factors. Notably, it has been reported that the origin-bound Fkh1 proteins can interact with each other by the domain-swapping mechanism which is necessary for the clustering of origins in the cell nucleus (13). The region of Fkh1 that is essential for domain swapping is located inside its DNA binding domain, between amino acid residues 310 and 340. However, the domain-swapping mutant of Fkh1 binds replication origins as efficiently as the wild-type protein, indicating that the origin-binding activity of Fkh1 is not modulated by its domain-swapping properties (13).

The very precise spatial organization of Fkh1/2 binding sites in replication origins supports the idea that the origin-bound Forkhead proteins must be oriented in a conformation that allows interaction between their monomers. This could be optional for the recruitment of Forkhead proteins to gene promoters, where interactions with other transcription factors, for example through the FHA domain, could stabilize the binding. Alternatively, sequence variability of the Fkh1/2 binding sites may influence the affinity of Forkhead proteins for these sites. For example, it has been found that the site GTAAACA in the *SWI5* promoter is a “strong” binding site, whereas the sequence ATAAACA is a ‘weak’ site (31). Furthermore, the bioinformatics study of Fkh1/2 binding sites in the yeast genome has revealed that the *CLB2* cluster genes are significantly enriched for “strong” Forkhead binding sites, while Forkhead-activated origins are not (38). This difference may also explain why the activation of DNA replication origins by Fkh1 requires its dimerization-mediated stabilization, whereas it is dispensable for regulating *CLB2* cluster genes.

**Figure 6:**
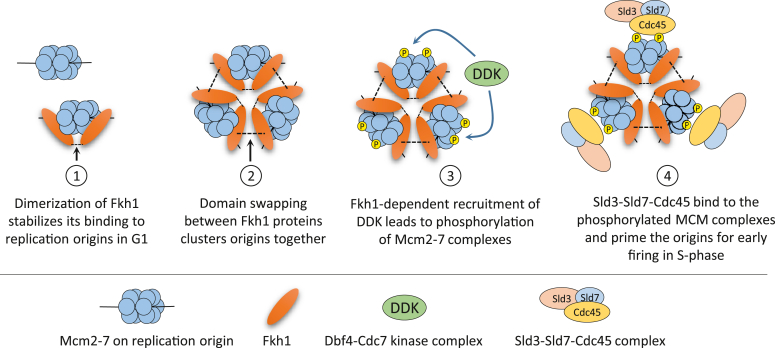
**Model of Fkh1-dependent regulation of early DNA replication origins based on our data as well as on the previous results by Ostrow *et al.*** (13) **and Fang *et al.*** (12).

Collectively, these data suggest that Forkhead-mediated protein-protein interactions are required at multiple levels for inducing the early firing of DNA replication origins. First, dimerization can stabilize the binding of Forkhead proteins to the origins. Second, the domain swapping mechanism can mediate the clustering of Forkhead-bound origins, and third, Forkhead proteins can recruit DDK kinase to the origins, leading to the phosphorylation of the MCM complex and the recruitment of Sld3-Sld7-Cdc45 proteins (Fig. 6).

## Experimental procedures

### Yeast strains, antibodies, immunoprecipitation, and anchor-away assay

All yeast strains were derivatives of *Saccharomyces cerevisiae* strain W303 (39) and are listed in Table S1. The sequences encoding either triple FLAG, 3F12, or 1E2 epitope tags were fused to the genes of DNA replication factors in their native loci, and the expression of the epitope-tagged proteins was confirmed by Western blot (Fig. S6). In strains expressing different mutants of Fkh1, the native *FKH1* allele was replaced with the mutant version of the gene in its original genomic location. Anti-FLAG antibody M2 (Sigma; F1804) and anti-E2 antibodies 3F12 and 1E2 (Icosagen) were used to detect and immunoprecipitate the tagged proteins. For Western analysis, 1 ml of exponentially growing cells were lysed as described in (40). For immunoprecipitations, cell pellets from 50 ml of exponentially grown cells were resuspended in 1 ml of lysis buffer (50 mM HEPES pH7.5, 150 mM NaCl, 1 mM EDTA, 10% glycerol, 0.5% Triton X-100, 0.5 mM DTT, and 1x Roche protease inhibitor cocktail) and mechanically sheared in the presence of 0.5 mm glass beads. After 5 min of centrifugation at 16,000*g*, 1 ml of supernatant (whole cell lysate, WCL) was incubated with 1 μg of anti-FLAG M2 or 5E11 antibodies at 4 °C for 1.5 h. 20 μl of rProtein A Sepharose Fast Flow beads (Merck; GE17-1279-01) were added, and WCL was further incubated at 4 °C for 1.5 h. Beads were washed three times with WB1 (50 mM HEPES pH 7.5, 150 mM NaCl, 1 mM EDTA, 5% glycerol, 0.5% Triton X-100, 0.5 mM DTT) and once with WB2 (50 mM HEPES pH 7.5, 150 mM NaCl, 1 mM EDTA, 5% glycerol). Beads were resuspended in 25 μl 2XSDS sample buffer (125 mM Tris-HCl, pH 6.8, 4% SDS, 10% 2-mercaptoethanol, 20% glycerol, 0,004% bromophenol blue), incubated for 5 min at 95 °C, and vortexed briefly. Western blot samples and immunoprecipitated proteins were separated on 8%, 10%, or 15% SDS-polyacrylamide gels and detected by immunoblotting using anti-FLAG or anti-E2 primary antibodies with a dilution of 1:10,000. Anti-mouse HRP-conjugated secondary antibody (1:15,000) and Immobilon Western Chemiluminescent HRP substrate (Millipore; WBKLS0500) were used for signal visualization on ChemiDocXRS^+^/ImageLab Software (BioRad). In the strains used for conditional depletion of proteins from the cell nucleus, the designated proteins were C-terminally tagged with the FKBP12-rapamycin-binding (FRB) domain of the human mTOR protein, and the FKBP12 domain was fused with the ribosomal protein RPL13A (29). Depletion of proteins was induced with rapamycin (Cayman Europe), and inhibition of the strain growth was confirmed by the spot test assay (Fig. S1*B*), as described previously (41, 42).

### Cell cycle arrest, chromatin immunoprecipitation, DNA doubling assay, and RT-qPCR

For the arrest of cells in G1, 100 nM α-factor mating pheromone (Zymo Research) was added to the growth medium for 3 h before the collection of cells. The G1-arrest of strains was assessed microscopically by detecting shmoo formation in the majority of cells and by flow cytometry analysis. Chromatin immunoprecipitation (ChIP) assays were performed as described previously (28). Shortly, whole-cell extract from 8 × 10^7^ cells was used for ChIP assays with 0.5 μg of anti-E2 tag or anti-FLAG antibody or with no antibody as a control. Co-precipitated DNA was analyzed by quantitative PCR (qPCR) using the Roche Lightcycler 480 real-time PCR system under standard conditions (40 cycles; 95 °C for 15 s, 58 °C for 20 s, and 72 °C for 20 s). The ChIP signals were normalized to the no antibody reaction, which was considered the background and was arbitrarily given value 1. For comparison of Fkh1-dependent and -independent recruitment of pre-RC factors to replication origins, the test strains contained two copies of *ARS607* origins, a wild-type origin in its native locus, and an additional copy of *ARS607* with the mutated 3′-Fkh1/2 binding site in the *VPS13* locus, as described previously (19). ChIP signals from both loci were detected by qPCR with primers specific for the unique sequences flanking the loci. For the genomic DNA doubling assay, the yeast strains were arrested in G1 for 3 h and then released into YPD media containing 200 mM hydroxyurea (HU) at 30 °C. Samples were collected after 120 min of release from G1, and the relative copy number of early-replicating *ARS305*, *ARS607*, and late-replicating *ARS522* was determined by qPCR as described previously (28). For the detection of mRNA, samples were collected 3 h after α-factor-mating pheromone addition and 60 min after the release from G1. Cells were collected, and RNA was extracted as described previously (43). DNaseI treatment was carried out, and 100 ng of RNA was used for one-step reverse transcriptase quantitative real-time PCR (RT-qPCR) using the LightCycler Real-Time RCR System (42 °C for 60 min, 95 °C for 10 min, and 40 cycles of 95 °C for 15 s, 55 °C for 1 min). Primers specific for the coding regions of Forkhead-regulated genes *CLB2*, *SWI5*, and *IRC8* were used. The results were normalized with the *FBA1* mRNA, which is highly and stably expressed throughout the cell cycle. Three biological repeats were performed for all ChIP, DNA doubling, and RT-qPCR experiments, each analyzed by three technical repeats. To validate the statistical significance of the results, the two-sample homoscedastic *t* test was used for the calculation of the *p*-values. The sequences of qPCR primers are listed in Table S2.

### Flow cytometry, microscopy, and cell sedimentation assay

For flow cytometry analysis of the cell cycle, 1 ml of yeast culture was fixed in 10 ml of ice-cold 70% ethanol. Samples were prepared and stained with SYBR Green I (Invitrogen) for flow cytometry analysis as described previously (41). Data from 10,000 cells were collected with FACS Aria (Becton Dickinson), and the cell cycle distribution was analyzed with Cyflogic software. For cell morphology analysis, exponentially growing yeast cells were collected and sonicated for 15 s before imaging using an Olympus BX61 microscope at 400× magnification. All images were collected with CellSens software and analyzed in ImageJ. For cell sedimentation assay, the strains expressing different Fkh1 deletion mutants were grown to the late-log phase and diluted to a concentration of 5 × 10^7^ cells per ml. 3 ml of the cell suspension was transferred to transparent tubes, placed on a vertical stand, and photographed at 15-min intervals for 2 h.

### Protein purification and SEC

Genes encoding N-terminally FLAG-tagged full-length and Δ412–428 Fkh1 proteins were cloned under the control of a galactose-inducible *GAL1-10* promoter and integrated into the *ADE2* locus in strains AKY2539 and AKY2540. 500 ml of yeast cultures (approximately 1 × 10^7^ cells per ml) were induced for 3 h in the galactose-containing growth medium, collected, washed with water, and snap-frozen in liquid nitrogen for later analysis. To prepare the extracts, the frozen pellets were crushed together with 3 ml of frozen ‘popcorn’ of 600N buffer (50 mM Hepes - NaOH pH 7.6, 600 mM NaCl, and 10% glycerol) supplemented with 0.1% Tween-20, 2 mM β-mercaptoethanol, and Roche protease inhibitor cocktail. Three 3-min bursts with a frequency setting of 30 s^−1^ were applied in an MM400 Retsch ball mill, cooling down the grinding jars with cells in liquid nitrogen between the bursts. The extract was thawed and its total volume was adjusted to 30 ml with an additional 600N lysis buffer before clearing with centrifugation at 50,000*g* for 30 min. 300 μl (packed volume) of anti-FLAG M2 agarose beads (Sigma Aldrich) were added to the cleared extract and incubated for at least 2 h at 4 °C in the rotating mixer. Beads were then collected by centrifugation, transferred into a disposable chromatography column, washed first three times with 600N buffer supplemented with 0.1% Tween-20, 1 mM β-mercaptoethanol, and 0.2 mM PMSF, then washed additionally twice with 150N buffer (25 mM Hepes - NaOH pH 7.6, 150 mM NaCl, 10% glycerol, and 1 mM β-mercaptoethanol). The proteins bound to the resin were eluted with 150N + 200 μg/ml FLAG peptide. For carrying out the SEC analysis, the flag eluate was concentrated in Amicon Ultra-4 centrifugal filter units before injecting it into the Superdex 200 5/150 GL SEC column attached to the GE Healthcare μÄKTA chromatography system. The column was developed with a 150N buffer at an elution speed of 50 μl/min, and 50 μl fractions were collected. To determine the Stokes radius of the purified Fkh1 proteins, the column was calibrated with the help of the following protein standards: carbonic anhydrase (Rs = 2.1), ovalbumin (Rs = 3.05), aldolase (Rs = 4.81), ferritin (Rs = 6.1), and thyroglobulin (Rs = 8.5).

### Sedimentation analysis of the purified Fkh1 proteins

4 ml of 10 to 15% linear glycerol gradients in a 25 mM HEPES-NaOH pH 7.6, 150 mM NaCl buffer were poured using a GE Healthcare μÄKTA chromatography system. Purified Fkh1 protein was mixed with the following protein standards with known Svedberg coefficients: aldolase (S20,w = 7.35), conalbumin (S20,w = 5.05), and ovalbumin (S20,w = 3.66) (44). 100 μl of the protein mix (with glycerol concentration diluted to 5%) was loaded on top of the gradient and centrifuged in a Beckman Optima L90-K ultracentrifuge in an SW55 swinging-bucket rotor at 180,000*g* for 18 h at 4 °C. 14 300 μl fractions were collected from the top of each gradient. Samples from the fractions were run in the 10% SDS-PAGE and then either silver stained for detecting the sedimentation standard proteins or transferred to the PVDF filter and probed with the anti-FLAG antibodies to detect Fkh1. Stained electrophoresis gels and Western blots were scanned and subjected to a densitometry analysis using Fiji software (45). The apparent molecular weight of Fkh1 was calculated from the Rs and S using the following simplified formula: Mw(app) = 4.205(SR) (46).

## Data availability

All data are presented within the manuscript and Supporting Information.

## Supporting information

This article contains supporting information (46).

## Conflict of interest

The authors declare that they have no conflicts of interest with the contents of this article.
