# Supplementary material for: Interactions between Fkh1 monomers stabilize its binding to DNA replication origins
Source: J Biol Chem. 2023 Jul 7;299(8):105026. doi: 10.1016/j.jbc.2023.105026 (PMC10403728; doi:10.1016/j.jbc.2023.105026)
Supplement: Supporting Table S1 [file mmc1.docx]

**Supplementary Table 1. Yeast strains**

| **Strain** | **Genotype** | **Source** |
| --- | --- | --- |
| AKY1440 | *W303; MAT A tor1-1 fpr1::LEU2 rpl13a::RPL13A-2xFKBP12-TRP1 cdc45::CDC45-3x1E2tag-spHIS5 fkh2::FKH2-FRB-kanMX6 bar1::hphMX6* | This study |
| AKY1446 | *W303; MAT A tor1-1 fpr1::LEU2 rpl13a::RPL13A-2xFKBP12-TRP1* | This study |
| AKY1477 | *W303; MAT A fkh1::FKH1-3x3F12tag-natMX6 cdc45::CDC45-3x1E2tag-spHIS5 fkh2::LEU2 bar1::hphMX6* | This study |
| AKY1478 | *W303; MAT A fkh1::fkh1Δ2-22-3x3F12tag-natMX6 cdc45::CDC45-3x1E2tag-spHIS5 fkh2::LEU2 bar1::hphMX6* | This study |
| AKY1484 | *W303; MAT A fkh1::fkh1Δ410-484-3x3F12tag-natMX6 cdc45::CDC45-3x1E2tag-spHIS5 fkh2::LEU2 bar1::hphMX6* | This study |
| AKY1567 | *W303; MAT A fkh1::3xFlag-FKH1-natMX6 cdc45::CDC45-3x1E2tag-spHIS5 fkh2::klURA3 bar1::hphMX6* | This study |
| AKY1568 | *W303; MAT A fkh1::3xFlag- fkh1Δ471-484-natMX6 cdc45::CDC45-3x1E2tag-spHIS5 fkh2::klURA3 bar1::hphMX6* | This study |
| AKY1569 | *W303; MAT A fkh1::3xFlag- fkh1Δ429-484-natMX6 cdc45::CDC45-3x1E2tag-spHIS5 fkh2::klURA3 bar1::hphMX6* | This study |
| AKY1570 | *W303; MAT A fkh1::3xFlag- fkh1Δ410-484-natMX6 cdc45::CDC45-3x1E2tag-spHIS5 fkh2::klURA3 bar1::hphMX6* | This study |
| AKY1633 | *W303; MAT A vps13::GALpr-vps13-3kb-ARS607-B3mut-TRP1 cdc45::CDC45-3xFlag-natMX6 bar1::hphMX6* | This study |
| AKY1634 | *W303; MAT A vps13::GALpr-vps13-3kb-ARS607-B3mut-TRP1 dpb11::DPB11-3xFlag-natMX6 bar1::hphMX6* | This study |
| AKY1639 | *W303; MAT A vps13::GALpr-vps13-3kb-ARS607-B3mut-TRP1 psf2::PSF2-3xFlag-natMX6 bar1::hphMX6* | This study |
| AKY1640 | *W303; MAT A vps13::GALpr-vps13-3kb-ARS607-B3mut-TRP1 pol2::POL2-3xFlag-natMX6 bar1::hphMX6* | This study |
| AKY1654 | *W303; MAT A tor1-1 fpr1::LEU2 rpl13a::RPL13A-2xFKBP12-TRP1 cdc45::CDC45-3x1E2tag-spHIS5 fkh1::FKH1-3x3F12-natMX6 fkh2::FKH2-FRB-kanMX6 vps13::GALpr-vps13-3kb-ARS607-TRP1 bar1::hphMX6* | This study |
| AKY1667 | *W303; MAT A vps13::GALpr-vps13-3kb-ARS607-B3mut-TRP1 sld3::SLD3-3xFlag-natMX6 bar1::hphMX6* | This study |
| AKY1668 | *W303; MAT A vps13::GALpr-vps13-3kb-ARS607-B3mut-TRP1 fkh1::FKH1-3xFlag-natMX6 bar1::hphMX6* | This study |
| AKY1688 | *W303; MAT A tor1-1 fpr1::LEU2 rpl13a::RPL13A-2xFKBP12-TRP1 cdc45::CDC45-3x1E2tag-spHIS5 fkh1::3xFlag-FKH1-FRB-natMX6 fkh2::FKH2-FRB-kanMX6 bar1::hphMX6* | This study |
| AKY1701 | *W303; MAT A tor1-1 fpr1::LEU2 rpl13a::RPL13A-2xFKBP12-TRP1 cdc45::CDC45-FRB-klURA3 fkh1::FKH1-3x3F12-natMX6 fkh2::FKH2-FRB-kanMX6 bar1::hphMX6* | This study |
| AKY1702 | *W303; MAT A tor1-1 fpr1::LEU2 rpl13a::RPL13A-2xFKBP12-TRP1 cdc45::CDC45-3x1E2tag-spHIS5 sld3::SLD3-FRB-klURA3 fkh1::FKH1-3x3F12-natMX6 fkh2::FKH2-FRB-kanMX6 bar1::hphMX6* | This study |
| AKY1741 | *W303; MAT A vps13::GALpr-vps13-3kb-ARS607-B3mut-TRP1 cdc7::CDC7-3xFlag-natMX6 bar1::hphMX6* | This study |
| AKY1743 | *W303; MAT A tor1-1 fpr1::LEU2 rpl13a::RPL13A-2xFKBP12-TRP1 cdc45::CDC45-3x1E2tag-spHIS5 sld7::SLD7-FRB-klURA3 fkh1::FKH1-3x3F12-natMX6 fkh2::FKH2-FRB-kanMX6 bar1::hphMX6* | This study |
| AKY1746 | *W303; MAT A tor1-1 fpr1::LEU2 rpl13a::RPL13A-2xFKBP12-TRP1 cdc45::CDC45-3x1E2tag-spHIS5 pol2::POL2-FRB-klURA3 fkh1::FKH1-3x3F12-natMX6 fkh2::FKH2-FRB-kanMX6 bar1::hphMX6* | This study |
| AKY1756 | *W303; MAT A vps13::GALpr-vps13-3kb-ARS607-B3mut-TRP1 dbf4::DBF4-3xFlag-natMX6 bar1::hphMX6* | This study |
| AKY1773 | *W303; MAT A vps13::GALpr-vps13-3kb-ARS607-B3mut-TRP1 sld7::SLD7-3xFlag-natMX6 bar1::hphMX6* | This study |
| AKY1779 | *W303; MAT A fkh1::3xFlag-fkh1Δ410-428-natMX6 cdc45::CDC45-3x1E2tag-spHIS5 fkh2::klURA3 bar1::hphMX6* | This study |
| AKY1780 | *W303; MAT A fkh1::3xFlag-fkh1Δ417-428-natMX6 cdc45::CDC45-3x1E2tag-spHIS5 fkh2::klURA3 bar1::hphMX6* | This study |
| AKY1781 | *W303; MAT A fkh1::3xFlag-fkh1Δ423-428-natMX6 cdc45::CDC45-3x1E2tag-spHIS5 fkh2::klURA3 bar1::hphMX6* | This study |
| AKY1782 | *W303; MAT A fkh1::3xFlag-fkh1Δ412-428-natMX6 cdc45::CDC45-3x1E2tag-spHIS5 fkh2::klURA3 bar1::hphMX6* | This study |
| AKY1788 | *W303; MAT A tor1-1 fpr1::LEU2 rpl13a::RPL13A-2xFKBP12-TRP1 cdc45::CDC45-3x1E2tag-spHIS5 dbf4::DBF4-FRB-klURA3 fkh2::FKH2-FRB-kanMX6 bar1::hphMX6* | This study |
| AKY1789 | *W303; MAT A tor1-1 fpr1::LEU2 rpl13a::RPL13A-2xFKBP12-TRP1 cdc45::CDC45-3x1E2tag-spHIS5 cdc7::CDC7-FRB-klURA3 fkh2::FKH2-FRB-kanMX6 bar1::hphMX6* | This study |
| AKY1797 | *W303; MAT A tor1-1 fpr1::LEU2 rpl13a::RPL13A-2xFKBP12-TRP1 cdc45::CDC45-3x1E2tag-spHIS5 sld7::SLD7-FRB-klURA3 fkh2::FKH2-FRB-kanMX6 bar1::hphMX6* | This study |
| AKY1798 | *W303; MAT A tor1-1 fpr1::LEU2 rpl13a::RPL13A-2xFKBP12-TRP1 cdc45::CDC45-3x1E2tag-spHIS5 sld3::SLD3-FRB-klURA3 fkh2::FKH2-FRB-kanMX6 bar1::hphMX6* | This study |
| AKY1799 | *W303; MAT A tor1-1 fpr1::LEU2 rpl13a::RPL13A-2xFKBP12-TRP1 cdc45::CDC45-3x1E2tag-spHIS5 psf2::PSF2-FRB-klURA3 fkh2::FKH2-FRB-kanMX6 bar1::hphMX6* | This study |
| AKY2539 | *W303; MAT alpha LYS2 pep4::kanMX6 ade2::GALpr-fkh1Δ412-428-3xFlag-ADE2* | This study |
| AKY2540 | *W303; MAT alpha LYS2 pep4::kanMX6 ade2::GALpr-FKH1-3xFlag-ADE2* | This study |
