# Supplementary material for: Interactions between Fkh1 monomers stabilize its binding to DNA replication origins
Source: J Biol Chem. 2023 Jul 7;299(8):105026. doi: 10.1016/j.jbc.2023.105026 (PMC10403728; doi:10.1016/j.jbc.2023.105026)
Supplement: Supporting Table S2 [file mmc2.docx]

**Supplementary Table 2. qPCR primers**

| **Target locus** | **Primer name** | **Sequence 5’->3’** |
| --- | --- | --- |
| ARS607 | ARS607_F1_KJ | GGCTCGTGCATTAAGCTTGTA |
| ARS607 | ARS607_R_KJ | CAATAGCAGGATCGACCTGACT |
| VPS13_3kb | VPS3k_dist_F | TGATTCTATAAAGCTGGCAACGT |
| VPS13_3kb | VPS3k_dist_R | CTAAATACCGAATCCCTGGAAAA |
| ARS522 | ARS501_nII_F | ATTGAGCATTACCTAACGCCATA |
| ARS522 | ARS501_nII_R | TGGATCTGAAACCGAGCAGTT |
| ARS305 | ARS305_F_NAO5 | GCAGTGCTTGTAACTGGTGC |
| ARS305 | ARS305_R_NAO5 | TAGTTATTACGGCGTCGGGC |
| CLB2 promoter | CLB2_Prom_F | CCGCCAAAAGACAGATTTTATTC |
| CLB2 promoter | CLB2_Prom_R | ATATCGCGAACTTCGTTGTTGA |
| CLB2 coding region | Clb2-qPCR-F1 | CCTAAAAGGCGTTGGATCAA |
| CLB2 coding region | Clb2-qPCR-R1 | TTTGGGCAGTTCTTGTTCAA |
| SWI5 coding region | Swi5-qPCR-F1 | ACCAAAGAAAATTCGCTCGA |
| SWI5 coding region | Swi5-qPCR-R1 | CGCTTTCCTGATCTTCAACA |
| IRC8 coding region | Irc8-qPCR-F1 | ACAAAACTCCCCATGATTCG |
| IRC8 coding region | Irc8-qPCR-R1 | GGGTATCAATGGTGTTGCTT |
| FBA1 coding region | FBA1-REAL-D1F | TTGCACCCAATCTCTCCAAACTT |
| FBA1 coding region | FBA1-REAL-D1R | ATTTCTGGTCTCAAAGCGATGTCA |
