# Supplementary material for: Interactions between Fkh1 monomers stabilize its binding to DNA replication origins
Source: J Biol Chem. 2023 Jul 7;299(8):105026. doi: 10.1016/j.jbc.2023.105026 (PMC10403728; doi:10.1016/j.jbc.2023.105026)
Supplement: Supporting Figure S1 [file mmc3.pdf]

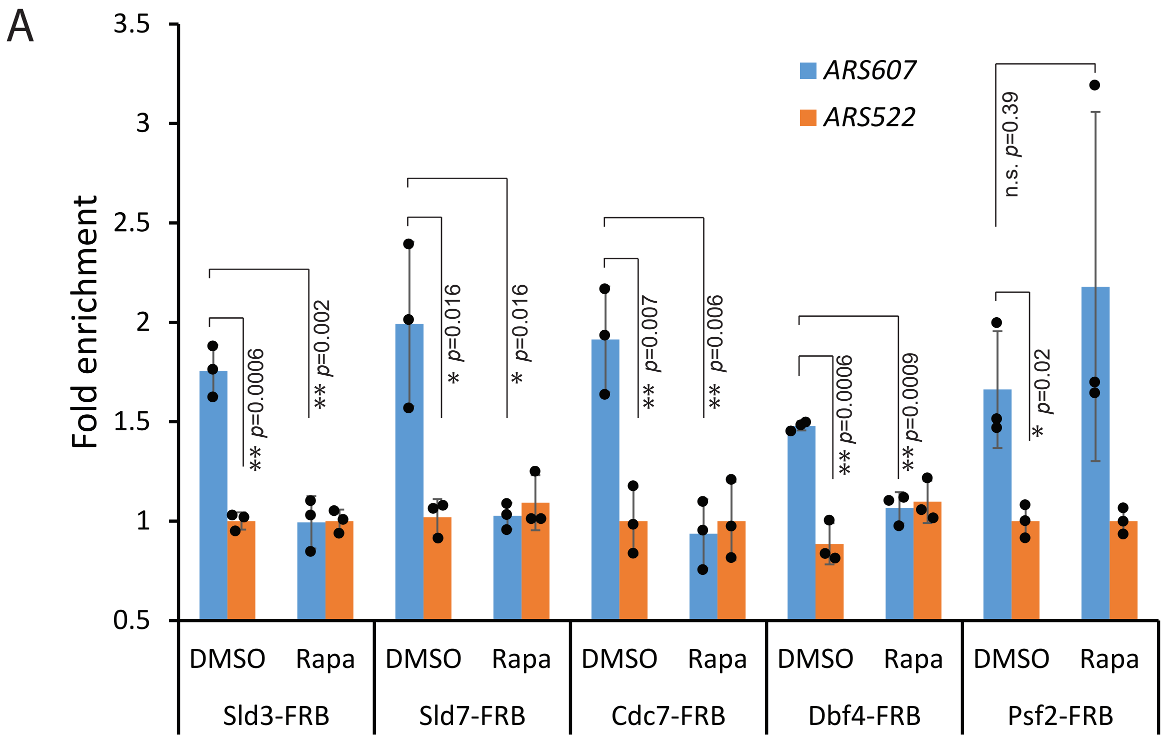

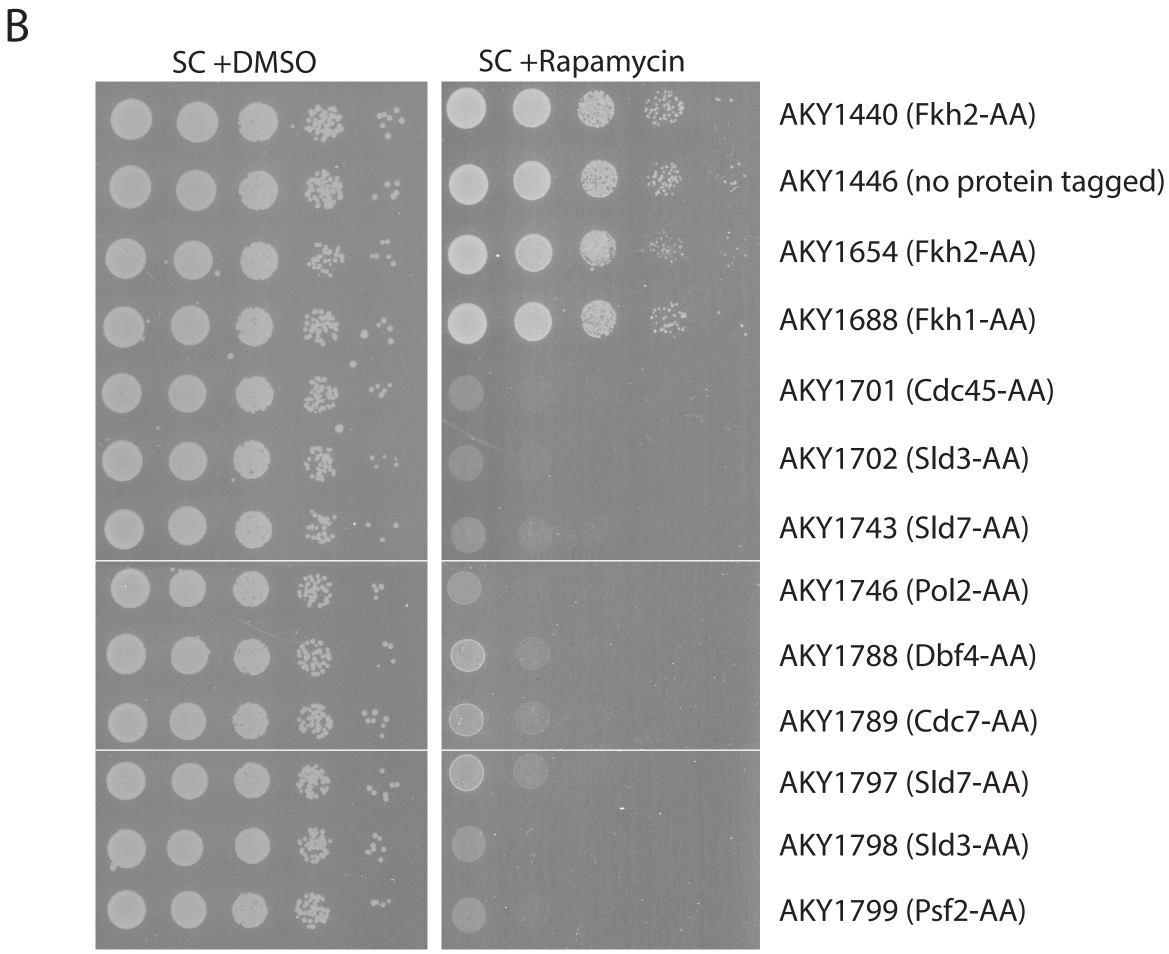

Figure S1

(A) DDK-dependent factors are required for the recruitment of Cdc45 to ARS607 in G1- arrested cells. The anchor-away strains expressing FRB tag in the C-terminus of Sld3, Sld7, Cdc7, Dbf4, or Psf2 proteins were arrested in G1, treated with rapamycin (Rapa) for the depletion of designated proteins, or with DMSO for control, and the presence of Cdc45 was detected on ARS607 and ARS522 loci. The graphs show the fold enrichment of the Cdc45 ChIP signal relative to no antibody control and represent the averages of three independent experiments. Black dots represent the individual data points, error bars represent the standard deviation. * indicates the p value < 0.05, ** indicates the p value < 0.01. n.s. = not significant.

(**B)** Validation of anchor-away (AA) strains used in this study. Ten-fold dilution series of the strains expressing the indicated proteins with C-terminal FRB-tag were seeded on a single plate containing either synthetic complete (SC) media supplemented with rapamycin (1µg/ml), or with DMSO as a control. Plates were incubated at 30°C for two days and photographed. For clearer presentation of the data, different parts of the single photo were rearranged into columns "SC +DMSO" and "SC +Rapamycin".
