# Supplementary material for: Interactions between Fkh1 monomers stabilize its binding to DNA replication origins
Source: J Biol Chem. 2023 Jul 7;299(8):105026. doi: 10.1016/j.jbc.2023.105026 (PMC10403728; doi:10.1016/j.jbc.2023.105026)
Supplement: Supporting Figure S2 [file mmc4.pdf]

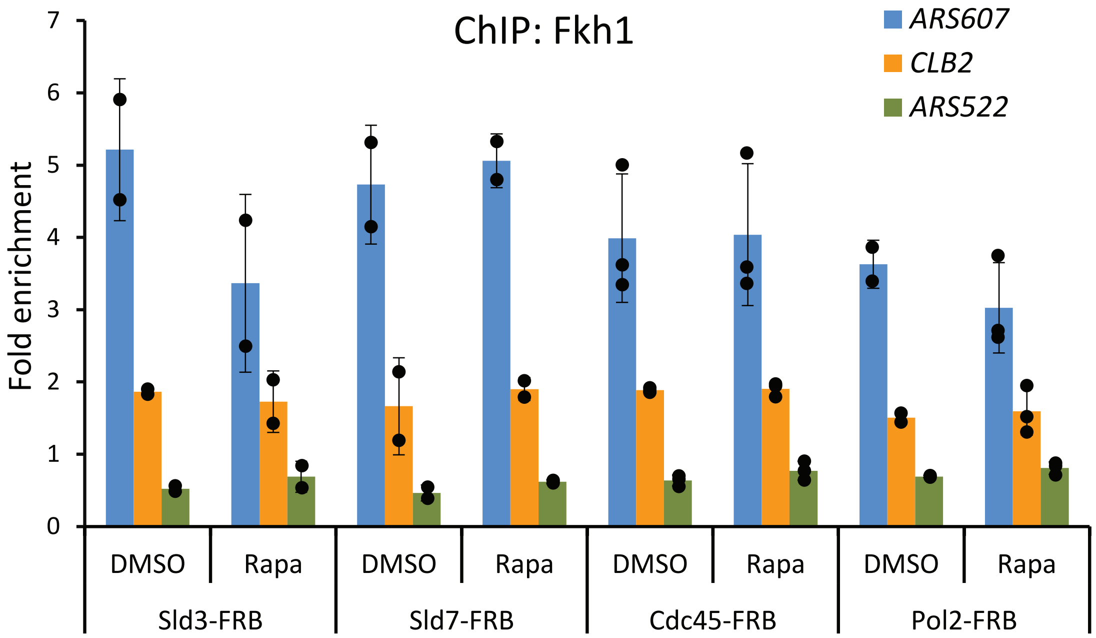

Figure S2. DDK-dependent factors are not required for the recruitment of Fkh1 to *ARS607* in G1-arrested cells. The anchor-away strains expressing FRB tag in the C-terminus of Sld3, Sld7, Cdc45, or Pol2 proteins were arrested in G1, treated with rapamycin (Rapa) for the depletion of designated proteins, or with DMSO for control, and the presence of Fkh1 was detected on *ARS607, ARS522,* and *CLB2* loci. The graphs show the fold enrichment of the Fkh1 ChIP signal relative to no antibody control and represent the average of three independent experiments. Black dots represent the individual data points, error bars represent the standard deviation.
