# Supplementary material for: Interactions between Fkh1 monomers stabilize its binding to DNA replication origins
Source: J Biol Chem. 2023 Jul 7;299(8):105026. doi: 10.1016/j.jbc.2023.105026 (PMC10403728; doi:10.1016/j.jbc.2023.105026)
Supplement: Supporting Figure S3 [file mmc5.pdf]

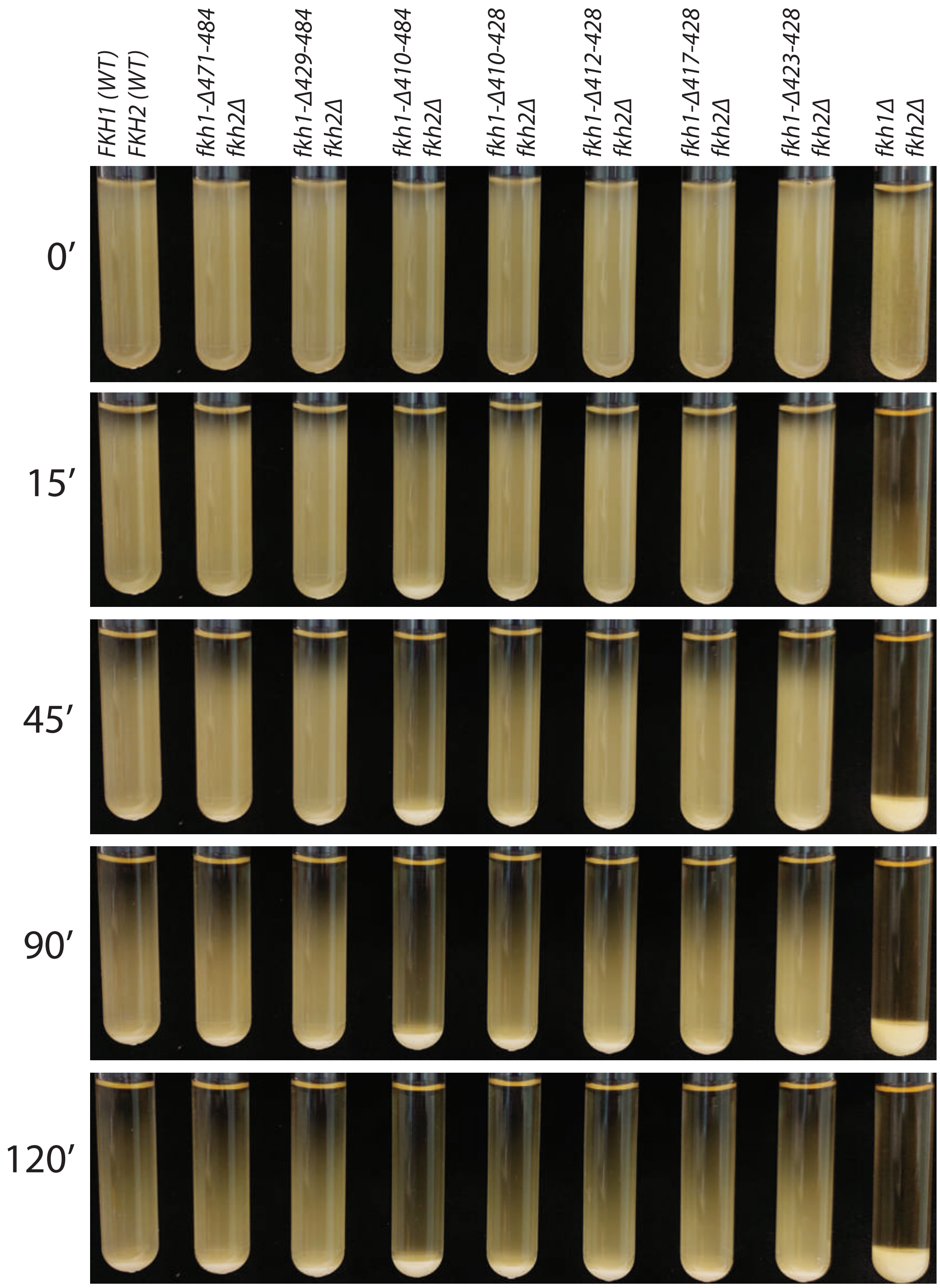

Figure S3

Sedimentation of Fkh1 C-terminal truncation and internal deletion strains. The strains expressing different deletion mutants of Fkh1 were grown to late-log phase and diluted to concentration 5E+07 cells per ml. 3ml of the cell suspension was transferred to transparent tubes and placed on a vertical stand. Tubes were photographed at time-points indicated on left, deletion mutants of Fkh1 are indicated on top. All strains except wild type were also *fkh2Δ*.
