# Supplementary material for: Interactions between Fkh1 monomers stabilize its binding to DNA replication origins
Source: J Biol Chem. 2023 Jul 7;299(8):105026. doi: 10.1016/j.jbc.2023.105026 (PMC10403728; doi:10.1016/j.jbc.2023.105026)
Supplement: Supporting Figure S4 [file mmc6.pdf]

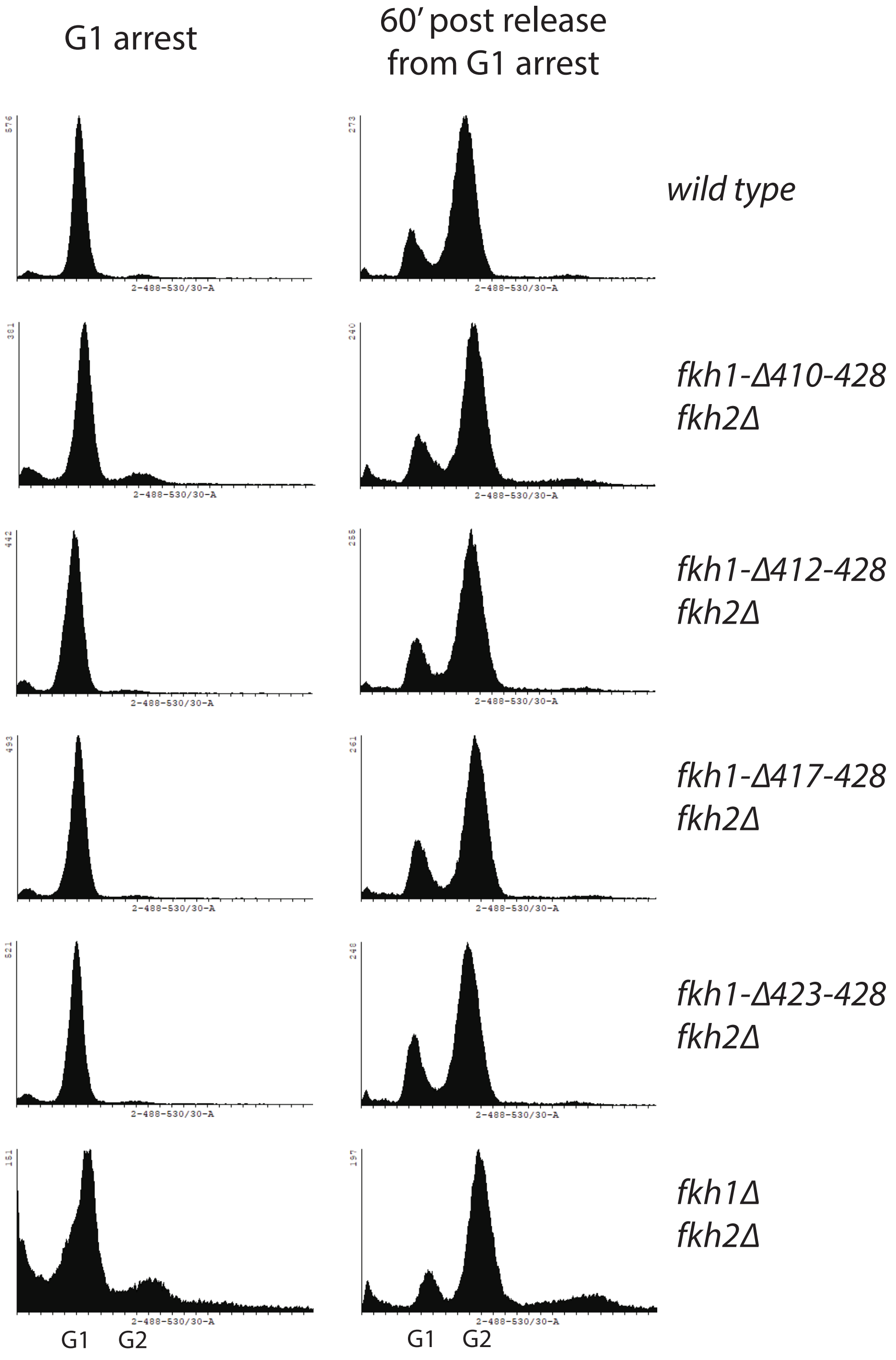

Figure S4

Cell cycle profiles (cell count vs DNA content) of Fkh1 internal deletion strains. The strains expressing different deletion mutants of Fkh1 were arrested in G1 with α-factor (left panels) and released from the arrest for 60 minutes (right panels). At 60 minutes time point, when the majority of cells were in the G2/M phase, samples were collected and mRNA levels of the Forkhead response genes were determined by RT-qPCR (Fig. 4C in the main article).
