# Supplementary material for: Interactions between Fkh1 monomers stabilize its binding to DNA replication origins
Source: J Biol Chem. 2023 Jul 7;299(8):105026. doi: 10.1016/j.jbc.2023.105026 (PMC10403728; doi:10.1016/j.jbc.2023.105026)
Supplement: Supporting Figure S5 [file mmc7.pdf]

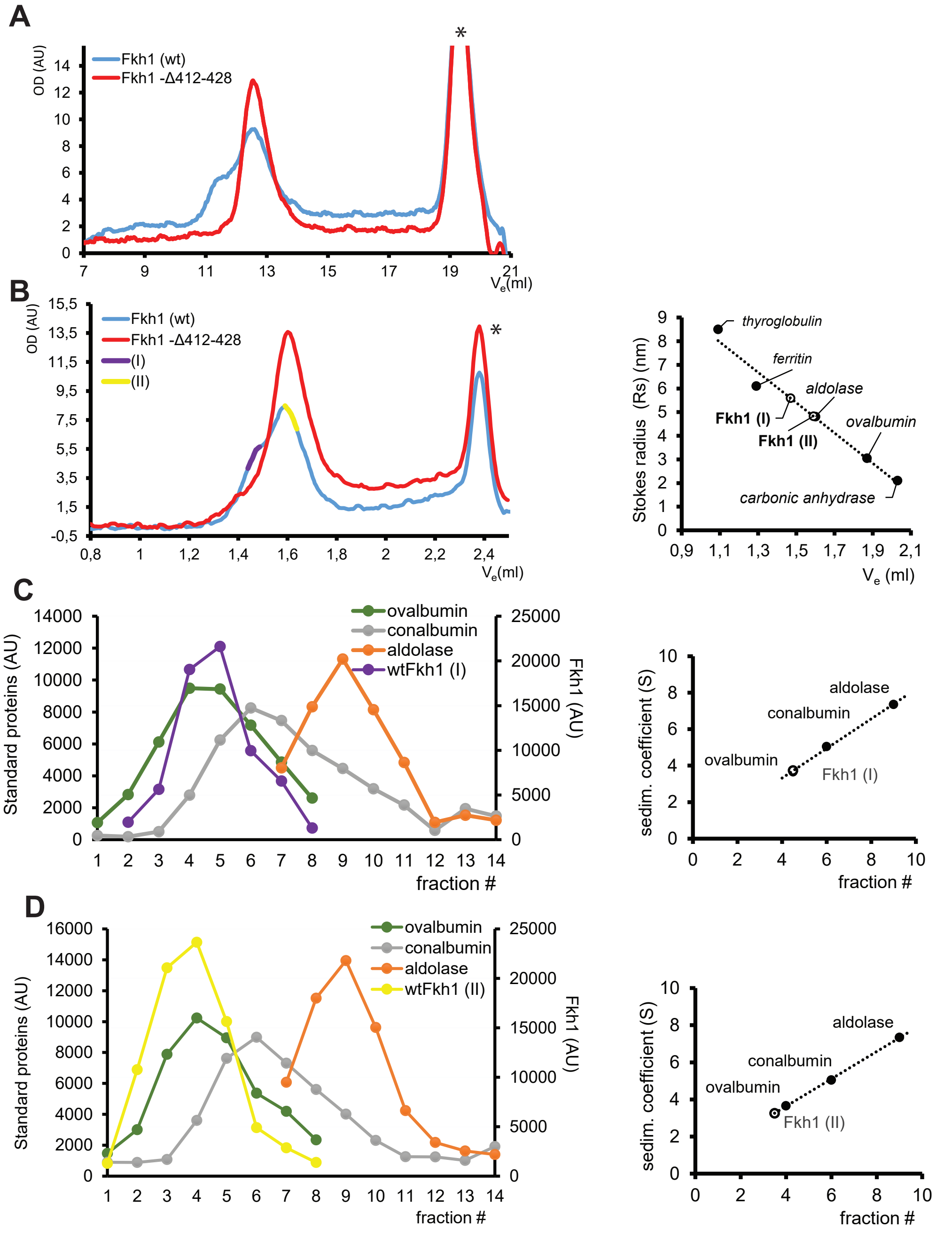

E

	S	Rs (nm)	Mw (kDa) calculated	Mw (kDa) expected monomer	Mw (kDa) expected dimer
I	3,7	5,6	88	58	116
II	3,3	4,8	66	58	116

Figure S5. Hydrodynamic analysis of the Fkh1 proteins.
(A)-(B) Chromatography profiles from independent size exclusion chromatography (SEC) runs with affinity purified wt and Δ 412-428 Fkh1 proteins, showing the consistency of the additional 'shoulder' in the wt protein peak. Superdex 200 10/300 GL or 5/150 GL columns were used in the runs shown on (A) and (B), respectively. Asterisk marks the additional Flag peptide peaks. Right graph in (B) plots the Stokes radiuses (Rs) of the wt Fkh1 'shoulder' (I) and 'main peak' (II) derived from the respective elution volumes (Ve) with the help of the calibration curve based on the elution volumes of indicated standard proteins with known Rs (see Materials and Methods for the details).

(C)–(D) Glycerol gradient sedimentation analysis of wt Fkh1 protein from the 'shoulder' (C) and 'main peak' (D) fractions of SEC. The 'shoulder' and the 'main peak' protein fractions used in this analysis were derived from the wt Fkh1 SEC run shown on (B) (marked by violet and yellow on the chromatography profile in (B), or as (I) and (II) in all the graph legends, respectively). Left graphs show the density profiles of Fkh1 and standard proteins in the gradient fractions, derived from the densitometry analysis of the proteins in the gradient fractions. The graphs on right present the calibration curves and estimated sedimentation coefficients of the Fkh1 proteins derived from this data (see Materials and Methods for the details).

(E) The values of the determined Stokes radiuses (Rs) and Svedberg sedimentation coefficients (S) of the Fkh1 proteins in the 'shoulder' and 'main peak' fractions together with the calculated molecular weight (Mw) of the respective proteins. The expected Mw values in the case of monomeric and dimeric Fkh1 protein are also shown in the table. Apparent Mw of the Fkh1 was calculated from the Rs and S using the following simplified formula: Mw(app) = 4.205(SR) (Erickson, 2009).

Erickson, H.P. (2009) Size and Shape of Protein Molecules at the Nanometer Level Determined by Sedimentation, Gel Filtration, and Electron Microscopy. *Biol Proced Online* **11**:32
