# Supplementary material for: Interactions between Fkh1 monomers stabilize its binding to DNA replication origins
Source: J Biol Chem. 2023 Jul 7;299(8):105026. doi: 10.1016/j.jbc.2023.105026 (PMC10403728; doi:10.1016/j.jbc.2023.105026)
Supplement: Supporting Figure S6 [file mmc8.pdf]

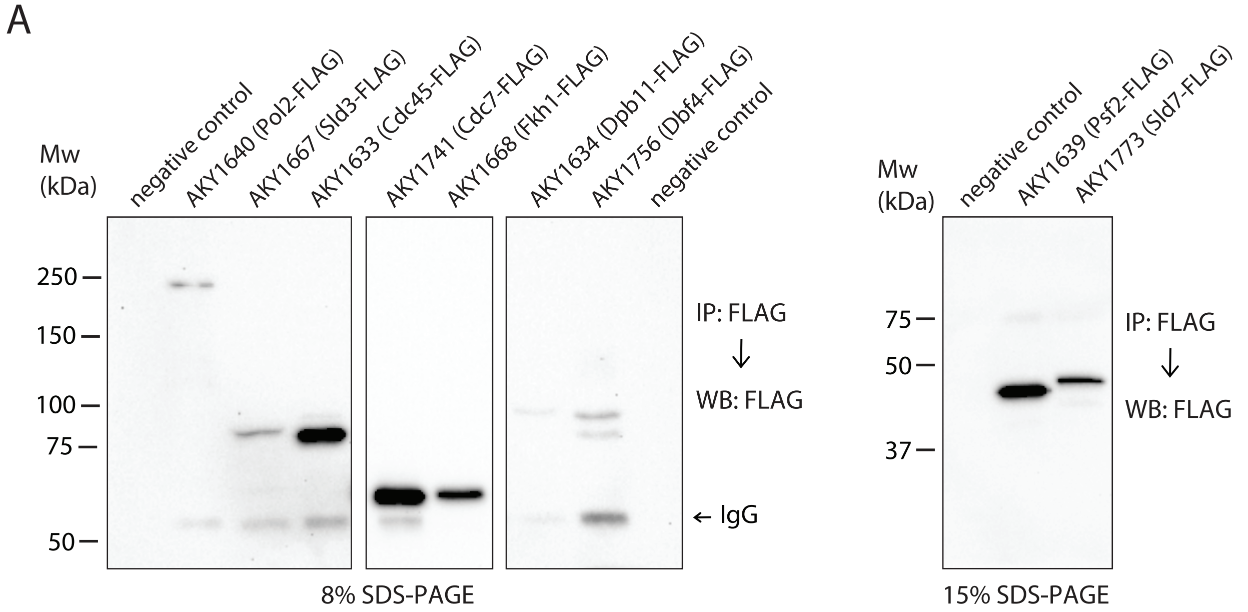

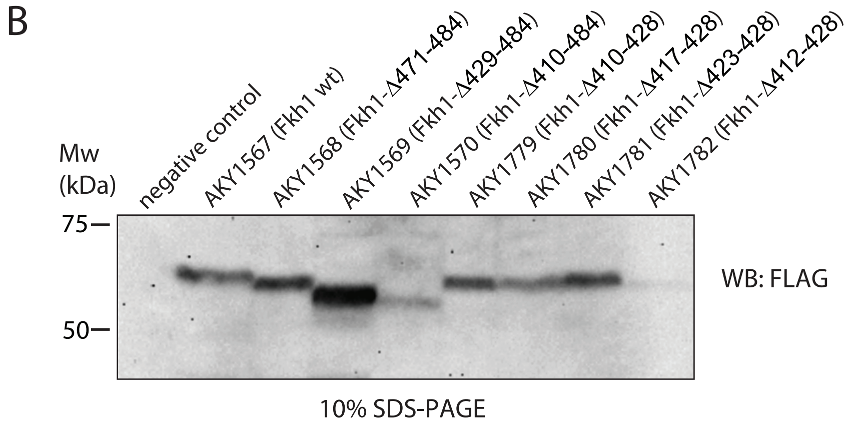

Figure S6. Verification of tagged protein expression in yeast strains.
(A) IP-western blot of tagged proteins from the indicated strains. The strains were grown to mid-log phase (1x10E7 cells per ml). 50 ml of the culture from each strain was used for preparation of the whole cell extract. The tagged proteins were first immunoprecipitated and then analyzed by western blot. 8% SDS-PAGE was used for separation of proteins with Mw >50kD (left panel), 15% SDS-PAGE was used for proteins <50 kD (right panel). Due to uneven endogenous expression levels of the replication factors, different exposure times of the blot are shown for different proteins in the left panel.
(B) Western blot of FLAG-tagged Fkh1 proteins from the strains indicated on top. The strains were grown to mid-log phase, 1x10E7 cells from each strain were collected and lysed. The protein samples were separated on 10% SDS-PAGE, followed by western blot with anti-FLAG antibody.
